# Revisiting Thymus Function

**DOI:** 10.3389/fimmu.2014.00411

**Published:** 2014-08-28

**Authors:** Jacques F. A. P. Miller

**Affiliations:** ^1^Department of Immunology, Walter and Eliza Hall Institute of Medical Research, Parkville, VIC, Australia

**Keywords:** thymus, thymic-dependent lymphocytes, adaptive immunity, cellular immunity, neonatal thymic function

For centuries, the thymus has been an organ in search of a function. The fact that it is a large mass of tissue in infancy was not appreciated at the beginning of the twentieth Century, as autopsies performed in infants succumbing to fatal illnesses such as diphtheria, revealed a small thymus. This resulted from stress during the illness, but the small size of the thymus was thought to be the norm. When infant death occurred during anesthesia for stress-unrelated conditions, fatality was blamed not on the anesthetic but on the large thymus. Some doctors even prescribed radiation therapy to shrink the thymus (1), not realizing that some of their patients would later develop adenocarcinoma of the thyroid.

Prior to 1961, the thymus was considered not to have any role in immunity. The major reasons for this can be summed up as follows. Unlike lymphocytes obtained by thoracic duct cannulation or from spleen and lymph nodes, thymus lymphocytes were generally poor in their ability to initiate immune reactions after adoptive transfer to appropriate recipients. Thoracic duct lymphocytes could home from blood into lymphoid tissues, “the only exception” being “the thymus in which very few small lymphocytes” appeared “to lodge” (2). The production of antibody-forming plasma cells and the formation of germinal centers, so prominent in spleen and lymph nodes, were not seen in thymus tissue of normal or immunized animals. Defects in immune responsiveness had never been documented in mice whose thymuses had been removed during adult life, a fact that had led some groups to conclude that “the thymus gland does not participate in the control of the immune response” (3). At a Symposium on Cellular Aspects of Immunity (4), in which took part world-renowned immunologists including Burnet, Good, Lederberg, Medawar, and Mitchison, and published in 1960, not a single reference was made to the thymus or to its cells throughout the meeting. Immunologists believed that, as a predominantly epithelial organ, the thymus had become vestigial during evolution and was just a graveyard for dying lymphocytes. Medawar even stated, “We shall come the regard the presence of lymphocytes in the thymus as an evolutionary accident of no very great significance” (5).

In the late 1950s, I was working on mice with lymphocytic leukemia that was induced in low-leukemic strain mice [as demonstrated by Ludwik Gross (6)] by injecting filtered extracts of leukemic tissues obtained from high leukemic strain mice. A leukemogenic virus was believed to be the causative agent and it had to be given to newborn mice to obtain a high incidence of leukemia. The disease began in the thymus and thymectomy at 1 month of age prevented its onset (7). Grafting a neonatal thymus 6 months after thymectomy restored the potential for leukemia development (8), and the virus could be recovered from the non-leukemic tissues of thymectomized mice (9). But why did it have to be given at birth? One possibility was that it could multiply only in neonatal thymus and would then spread to other sites. To test this, mice were thymectomized before the virus was given and therefore at birth.

The survivors grew well at first but, after weaning, many wasted and died prematurely whether inoculated with virus or not. Adult thymectomy, on the other hand, had never shown any untoward effects such as weight loss or obvious pathology. This led me to conclude “that the thymus at birth may be essential to life” (10). Histological examination of the tissues of neonatally thymectomized mice revealed a marked deficiency of lymphocytes in the circulation and the lymphoid tissues and many wasted mice had liver lesions suggesting infection by some hepatitis virus (11, 12). At that time Gowans had shown that small lymphocytes were not short lived cells, as had been thought before, but immunologically competent cells with a long lifespan, recirculating from blood through lymphoid tissues into lymph and able to initiate immunological reactions when appropriately stimulated by antigen (13). Clearly, my neonatally thymectomized mice must have been immunodeficient, which accounted for their susceptibility to virus infections. I therefore tested their immune competence by grafting skin from allogeneic mice and from rats. The results were incredibly spectacular and published first in The Lancet in 1961 (11) and in greater detail in the Proc Roy Soc. (12). The mice failed to reject skin both from totally unrelated strains (“H-2-incompatible”) and from rats, and failed to do so even when grafted before the onset of wasting. Since both Gowans and Medawar had firmly established that rejection of foreign skin grafts was mediated by lymphocytes, and since my mice were deficient in lymphocytes following neonatal thymectomy, it was logical for me to conclude that the thymus was the source of immunologically competent lymphocytes, at least during the neonatal period. Contrary to the prevailing opinion, I postulated “during embryogenesis the thymus would produce the originators of immunologically competent cells many of which would have migrated to other sites at about the time of birth. This would suggest that lymphocytes leaving the thymus are specially selected cells” (11). I had therefore proposed the bold postulate that the thymus was the site responsible for the development of immunologically competent small lymphocytes.

The few neonatally thymectomized mice that did eventually reject allogeneic skin grafts were later grafted again with skin from the same donors but showed no evidence of a second set response (12). By contrast, neonatally thymectomized mice bearing well-established allogeneic skin rejected that skin rapidly when given intravenous lymphocytes from normal donors that had been immunized to skin of the same strain (12).

I tested the ability of my neonatally thymectomized mice to produce antibody to *Salmonella typhi* H antigen and found this to be impaired (12).

Grafting thymus tissue to neonatally thymectomized mice prevented immunological deficiency. Although implantation of syngeneic thymus tissue allowed these mice to develop a normal immune system, grafting a thymus derived from a foreign strain induced specific immune tolerance to the histocompatibility antigens of the donor. Thus, lymphocytes developing in the thymus in the presence of foreign cells must have been deleted [i.e., “selectively thymectomized” as I suggested (12)]. Hence, by implication, the thymus should be the site where self tolerance is imposed and where discrimination between self and non-self takes place.

Showing that cells from the thymus migrated into the lymphoid tissues was difficult at that time, since no markers had been found to identify cells from different locations. So I made use of the T6 mouse strain the cells of which could easily be identified at metaphase by the presence of 2 min chromosomes. Neonatally thymectomized F1 hybrid mice in which one parent was T6, were grafted with thymus from the other parental strain and immunized with skin from various donors. An analysis of the chromosome constitution of the cells in metaphase in the spleen showed that 15–20% had originated from the thymus graft (12).

My conclusions concerning the immunological function of the thymus were regarded with skepticism by the immunological community. For example, Medawar was not convinced as evident from a letter he sent to me in which he wrote: “I take it that the thymic tissue seen in fishes is wholly or predominantly epithelial, as its phylogenetic origin suggests. It is a matter of some interest that many organs, which seem to become redundant in the course of evolution undergo a sort of lymphocytic transformation” (14). Trivial criticisms abounded: what I had observed must surely have occurred only in the strain of mice that I had been using; my mice must have been in such poor health that any surgical trauma would prejudice their ability to reject skin grafts; whatever the thymus might have been doing in my mice, it could not possibly do in humans! At a Ciba Foundation Symposium on Tumor Viruses of Murine Origin held in Perugia in June 1961, the first international meeting where I presented my results, R.J.C. Harris, claimed the following: “Dr. Delphine Parrott in our laboratory has been thymectomizing day-old mice and there is at present no evidence that these animals are immunologically weaker than normal animals. They do not retain skin grafts; they are living and breeding quite normally. They do not die of laboratory infections” (15).

These criticisms did not last very long as I and several other researchers repeated, confirmed, and extended my results. It was evident, for example, that the adult thymus would still play a role in immunogenesis and this was shown when the rest of the lymphoid system was destroyed by total body irradiation and the mouse protected by an injection of bone marrow (16, 17). The adult thymectomized irradiated and marrow protected mice were crucial to our subsequent demonstration of the existence of two major lymphocyte subsets, T and B cells (18). An avalanche of work followed these early investigations.

## Conflict of Interest Statement

The author declares that the research was conducted in the absence of any commercial or financial relationships that could be construed as a potential conflict of interest.
